# Investigating the Role of Ethnicity and Religion or Spirituality on the Risk of Self-Harm in Children and Adolescents: A Systematic Literature Review

**DOI:** 10.1192/bjo.2022.203

**Published:** 2022-06-20

**Authors:** Lin John

**Affiliations:** Cardiff University, Cardiff, United Kingdom

## Abstract

**Aims:**

Around the world rates of self-harm vary, placing immense strain on health services. Approximately 20% of children and adolescents are thought to engage in self-harm. The systematic review aims to explore the characteristics, risks and protective factors of ethnicity and religion on self-harm in comparison to the general population. Better identification of risk factors can help professionals and local authorities develop intervention programs to mitigate the incidence of self-harm.

**Methods:**

The Population, Exposure, Outcome, Study design and setting (PEOS) was used as a framework to formulate the question for this systematic review. A literature search was conducted using EMBASE, MEDLINE and APA PsycInfo databases and all English articles published between 2010 and 2020 were screened against the inclusion and exclusion criteria.

**Results:**

Fourteen studies which met the criteria were identified and appraised using the Joanna Briggs Institute (JBI) critical appraisal checklist.

Unintentional injuries, sexual behaviours, adverse childhood experiences, health status and poverty alongside racial discrimination were associated with self-harm and or suicidal ideation in ethnically diverse populations. In African Americans, Hispanics and Whites, violence or physical altercation, illicit substance misuse, sadness and hopelessness increased the risk of self-harm and or suicidal ideation. The association of subtle forms of discrimination and suicidal ideation was noted to be statistically significant for African Americans, whereas for Latinx this was only marginally increased. Low mood and hopelessness in African American girls, substance misuse in American Indian youths, and aggression in the Caribbean cohort were also noted to present with increased self harm.

Adolescent's religiosity and parental monitoring had both a direct and an indirect role for suicidal ideation reduction. Religious importance and attendance at religious services by offspring and parents decreased self-harm in female adolescents more than males.

There was a wide heterogeneity in the population and factors reviewed in the different studies, hence pooling of data for meta-analysis of the quantitative studies was not appropriate to estimate prevalence or association between factors and characteristics of the population.

**Conclusion:**

This narrative synthesis provides evidence that minority ethnic groups have unique factors, which can increase the rate of self-harm. Religion or spirituality favours a protective role in self-harm or suicide but not for suicidal ideation, although there were only a limited number of articles exploring this.

Future studies should focus on defining the ethnic groups further and exploring this and religious factors on a wider scale using standardised parameters.

